# Improvement in insulin resistance and favourable changes in plasma inflammatory adipokines after weight loss associated with two months’ consumption of a combination of bioactive food ingredients in overweight subjects

**DOI:** 10.1007/s12020-012-9863-0

**Published:** 2012-12-28

**Authors:** Mariangela Rondanelli, Annalisa Opizzi, Simone Perna, Milena Faliva, Sebastiano Bruno Solerte, Marisa Fioravanti, Catherine Klersy, Cava Edda, Paolini Maddalena, Scavone Luciano, Ceccarelli Paola, Castellaneta Emanuela, Savina Claudia, Lorenzo Maria Donini

**Affiliations:** 1 Department of Applied Health Sciences, Section of Human Nutrition and Dietetics, Faculty of Medicine, University of Pavia, Azienda di Servizi alla Persona di Pavia, Servizio Endocrino Nutrizionale, Istituto di Riabilitazione “Santa Margherita”, Via Emilia 12, Pavia, Italy; 2 Department of Internal Medicine, Geriatrics and Gerontology Clinic, University of Pavia, “Istituto Santa Margherita”, Pavia, Italy; 3 Service of Biometry & Clinical Epidemiology, Fondazione IRCCS “Policlinico San Matteo”, Pavia, Italy; 4 Experimental Medicine Department, Medical Physiopathology, Food Science and Endocrinology Section, Food Science and Human Nutrition Research Unit, Sapienza University of Rome, Rome, Italy; 5 “Villa delle Querce” Clinical Rehabilitation Institute, Rome, Italy

**Keywords:** Epigallocatechin gallate, Capsaicins, Piperine, l-carnitine, Dietary supplement, Obesity, Inflammation, Leptin, Adiponectin, Insulin resistance

## Abstract

This randomized, double blind, placebo-controlled, 8 week trial assessed the efficacy on metabolic changes produced by a consumption of a combination of bioactive food ingredients (epigallocatechin gallate, capsaicins, piperine and l-carnitine) versus a placebo, as part of a therapeutic ‘lifestyle change’ diet, in 86 overweight subjects. Forty-one patients (2/14 F/M; age 43.7 ± 8.5; BMI 30.3 ± 3.5 kg/m^2^) were randomized to the supplemented group and 45 (29/16; age 40.7 ± 10.2; BMI 30.0 ± 2.7) to the control group. We observed that consumption of the dietary supplement was associated with a significantly greater decrease in insulin resistance, assessed by homostasis model assessment (*p* < 0.001), leptin/adiponectin ratio (*p* < 0.04), respiratory quotient (*p* < 0.008). LDL-cholesterol levels (*p* < 0.01). Moreover, statistically significant differences were recorded between the two groups in relation to urinary norepinephrine levels (*p* < 0.001). Leptin, ghrelin, C-reactive protein decreased and resting energy expenditure increased significantly in the supplemented group (*p* < 0.05, 0.03, 0.02 and 0,02 respectively), but not in the placebo group; adiponectin decreased significantly in the placebo group (0.001) but not in the supplemented group, although no statistical significance between the groups was elicited. BMI, fat mass (assessed by DXA) and vascular endothelial growth factor significantly decreased, whilst the resting energy expenditure/free fat mass significantly increased in both groups. In general, a greater change was recorded in the supplemented group compared to the placebo, although no statistically significant difference between the two groups was recorded. These results suggest that the combination of bioactive food ingredients studied might be useful for the treatment of obesity-related inflammatory metabolic dysfunctions.

## Introduction

The prevalence of individuals who are classified as overweight and obese is a primary health concern due to the relationship between obesity and various cardiovascular diseases (CVD) [1, 2] and associated comorbidities [1]. The scale of this obesity epidemic creates a pressing consumer need for the successful development of a dietary supplement with a beneficial action on weight control. The post-absorptive action of a number of ingredients has been shown to influence satiety [3] and substrate use or thermogenesis [4]; these ingredients include epigallocatechin gallate (EGCG) from *Camellia sinensis*, capsaicins from *Capsicum annuum longum*, piperine from *Piper nigrum L* and l-carnitine. Of these active ingredients, EGCG, a catechin found in abundance in green tea, is the most common, and has been found to increase satiety [5, 6] and energy expenditure, as well as increasing markers of lipolysis [7–12]. Studies have also suggested that EGCG may alter food digestibility [13, 14] and downregulate stearoyl-CoA desaturase (SCD1) gene expression [13], which may reduce adiposity, decrease lipid synthesis and increase liver fatty acid oxidation, as was found in SCD1 knockout mice [13, 15]. Furthermore, recent studies have also shown that EGCG directly inhibits vascular endothelial growth factor (VEGF) expression [16, 17]. Moreover, in humans, green tea consumption has been inversely correlated with oxidative damage and with the levels of inflammation markers [18, 19].

Extensive research over the past two decades has revealed that obesity is a proinflammatory disease [20], and increasing evidence identifies inflammation as the potential link between adipose tissue expansion and cardiometabolic complications [21]. In fact, obesity is now considered to be a condition that facilitates the development of a low-grade inflammatory state characterized by increased plasma levels of pro-inflammatory cytokines and cytokine-like proteins known as adipokines [22]. One of the consequences of this state of inflammation is the development of insulin resistance [23].

Several spices have been shown to exhibit activity against obesity through their antioxidant and anti-inflammatory mechanisms [24]. Of these, in addition to EGCG, capsaicin has been investigated most extensively as an antioxidant agent [25]. The use of capsaicin has therefore been investigated as a treatment for obesity and obesity-related metabolic diseases, as well as for its thermogenic activity [26–30] and its satiating effect [31, 32].

Piperine is an active component of black pepper that can effectively suppress lipid peroxidation [33, 34] and enhance the bioavailability of curcumin and other substances through the inhibition of drug-metabolizing enzymes in the liver [35].

Carnitine is an integral component in the transportation of long-chain fatty acids within the mitochondria for oxidation [36]. Supplemental carnitine has been shown to increase weight loss in animals [37] and to increase the oxidation of long-chain fatty acids in adults [38, 39].

*Allium sativa* is a highly appropriate ingredient for inclusion within a dietary supplement for the treatment of obesity-related pathologies, due to its wide range of significant biological activities (hypocholesterolemic, hypolipidemic, anti-hypertensive, anti-diabetic, anti-thrombotic and anti-hyperhomocysteinemia effects, which are typical of thiosulfinates and other organosulfur compounds) [40].

Studies have shown that the diet typically consumed by the Italian population is insufficient to prevent sub-clinical iodine deficiency. In fact, physicians often advise people to use iodine salt. *Fucus vesiculosus* can present a valid alternative, especially for those consuming low-sodium diets. The use of iodine to treat obesity can ensure that the total daily requirement is consumed and, consequently, can ensure optimal thyroid function [41].

Therefore, the purpose of this study was to examine the changes produced by ingesting a combination of bioactive food ingredients (EGCG, capsaicin, piperine, carnitine, *Allium sativa* and *Fucus vescicolosus*) for two months on: (1) body composition, assessed using dual energy X-ray absorptiometry (DXA) evaluation, as the primary end-point; (2) satiety control, calculated using a visual anologue scale and ghrelin assessment; (3) thermogenesis, calculated using resting energy expenditure (REE) and respiratory quotient (RQ) assessment; 4) serum markers of lipolysis [free fatty acids (FFA) and glycerol]; (5) adipokine release (leptin, adiponectin); (6) indices of inflammation [C-reactive protein (CRP) -PCR-] and insulin resistance [homostasis model assessment (HOMA)]; (7) angiogenesis marker release (-VEGF-); and (8) urinary noradrenalin. Moreover, the effect on health-related quality of life, evaluated using the Short-Form 36-Item Health Survey, was also assessed. The decision to use a combination of compounds was prompted by the concept of putting together interesting compounds with different activities on the various causes of obesity, in order to act on the multiple issues involved.

## Methods

### Participants

The study was carried out following approval by the Ethics Committee of the Department of Internal Medicine and Medical Therapy at the University of Pavia (Italy). Subjects provided their written consent to participate in the study. Healthy males and females ages 25–45 years, with a body mass index greater than 25 kg/m^2^ and less than 35 kg/m^2^, were eligible for the study. All subjects had to provide complete medical histories, and all underwent a physical examination, anthropometric assessment and routine laboratory tests. Individuals suffering from any hepatic or renal disease, diabetes, unstable CVD, uncontrolled hypertension, an eating disorder (diagnosed bulimia), active cancer or who had undergone surgery for weight loss were all excluded from the study. Moreover, patients were excluded from the study if they met the Diagnostic and Statistical Manual-IV (DSM-IV) criteria for a current diagnosis of major depressive disorder as determined by the Structured Clinical Interview for DSM-IV Axis 1 Disorders (SCID-1) [42].

Subjects were also excluded if they were using any type of medication for weight loss, or were pregnant or lactating, or if they had entered menopause. All participants agreed to refrain from participating in any other weight-loss programme. Alcohol intake, smoking habits and physical activity were recorded. Sedentary and non-smoking subjects, who did not drink more than 6 glasses of wine a week and did not drink hard liquor, were admitted to the study.

### Procedures and study design

After 12 h of fasting and abstinence from water since midnight, the subjects arrived at around 8:00 am, using motorised transportation, at the Endocrinology and Clinical Nutrition Unit of Azienda di Servizi alla Persona di Pavia at the University of Pavia (Italy) and at the Dietetic and Metabolic Unit, ‘Villa delle Querce’ Clinical Rehabilitation Institute in Rome, Italy.

Blood samples were taken for the routine analysis measurements and to measure leptin, adiponectin, ghrelin, insulin, glycerol and FFAs. The assessment of REE and RQ by indirect calorimetry, and the assessment of body composition by DXA and anthropometry were carried out in the fasting state at baseline.

On the same morning, the patients took the 24-h urine test for noradrenalin assessment. The same evaluations were assessed after two months’ ingestion of 2 capsules per day of the dietary supplement or the placebo. The subjects were randomly assigned to one of the 2 groups in a double-blind parallel study.

### Rating of satiety

Visual analogue scales (VAS) were used to assess appetite sensations. The scale was dotted with phrases describing the various degrees of hunger or satiety, but subjects were free to choose any point along the scale; the point chosen was defined as the Haber score [43]. In order to calculate the values of the area under the curve (AUC) in response to the treatment (with respect to ground), the VAS measurements for each day, over the entire study period, were considered in this calculation using the trapezoid method, after rescaling the score from 0 to 20. The test was performed every day before lunch time by all the subjects included in the study.

### Body composition measurement

Bone mineral density and body composition was measured at baseline and at 12 months by DXA, using a Lunar Prodigy DEXA (GE Medical Systems, Waukesha, WI) [44].

### Assessment of REE

Respiratory exchange measurements using indirect calorimetry (Deltatrac Monitor II MBM-200, Datex Engstrom Division, Instruments Corp. Helsinki, Finland) were used to estimate REE, adhering to the recommended measurement conditions [45]. REE was calculated from O_2_ and CO_2_ volumes—as well as from urine excretion nitrogen values—using the Weir formula, and expressed as kcal/day [46].

### Anthropometry, weight-loss programme and food intake

Nutritional status was assessed using anthropometric measurements at baseline and after 2 months in both groups. Body weight and height were measured and the body mass index (BMI) was calculated (kg/m^2^). Skinfold thicknesses (biceps, triceps, suprailiac, subscapular) were measured twice using a Harpenden skinfold caliper at 5 min intervals at each site, following a standardized technique [47]. Sagittal abdominal diameter was measured at the L_4–5_ level in the supine position and waist girth was also measured. Anthropometric variables were measured by a single investigator.

Body weight reduction was induced by a low-energy mixed diet (55 % carbohydrates, 30 % lipids and 15 % proteins) providing 600 kcal less than individually estimated energy requirements based on the measured REE. The energy content and macronutrient composition of the diets adhered to the nutritional recommendations of the American Diabetes Association [48, 49]. These diets were designed to achieve weight losses of 0.5–1 kg per week; this type of diet is considered to be a low-risk intervention [50]. Individual diet plans were drawn up for each subject by the research dietitian. To optimize compliance, dietary instructions were reinforced each week by the same research dietician. Each consultation included a nutritional assessment and weighing. A 3-day weighed-food record of 2 weekdays and 1 weekend day was performed before the study and during the last week of intervention. One-day weighed-food records were completed in weeks 2, 5 and 7. Low-energy diets and dietary records were analyzed using a food-nutrient database (Rational Diet, Milan, Italy). In order to assess compliance to the weight-reduction programme, a 24-h dietary summary was assessed by the nutritionist at the end of the study.

### Assessment of depressive symptoms and health-related quality of life

Depressive symptoms, assessed by the Beck depression inventory (BDI) [51] and health-related quality of life, assessed by the Short-Form 36-Item Health Survey (SF-36) [52], were evaluated at baseline and after 2 months in both groups.

### Biochemical analyses

Fasting venous blood samples were drawn between 08.00 and 10.00 a.m. with the subjects in a sitting position. Clinical Chemistry parameters were detected on the Roche Cobas Integra 400 plus analyzer (Roche Diagnostics, Basel, Switzerland), using specially-designed commercial kits provided by the manufacturer. In particular, total serum cholesterol, triacylglycerol, HDL-cholesterol, total proteins, total bilirubin, iron, glucose, uric acid, creatinine, transaminase alanine aminotransferase, aspartate aminotransferase and gamma glutamyl transferase were measured using enzymatic-colorimetric methods. LDL cholesterol was calculated according to the Friedewald formula [53] for those specimens with triacylglycerol levels less than 400 mg/dl (<4.5 mmol/l). The CRP was determined by Nephelometric High-Sensitivity CRP (Dade Behring, Marburg, Germany). Hemochrome was measured using a Coulter automated cell counter MAX-M (Beckman Coulter, Inc., Fullerton, USA). Serum insulin levels were measured on a Roche Elecsys 2010 analyzer (Roche Diagnostics, Basel, Switzerland) using dedicated commercial electrochemiluminescent immunoassays. Insulin resistance was evaluated using the HOMA [54] and Quantitative insulin-sensitivity check index (QUICKI) [55]. Serum concentrations of FFA and glycerol were determined by a quantitative colorimetric assay (BioAssay Systems, Hayward, CA). Plasma acylated and unacylated ghrelin levels were measured using an enzyme immunometric assay based on a double-antibody sandwich technique (BioVendor, Brno, CZECH REPUBLIC). Serum adiponectin levels were measured using an enzyme-linked immunosorbent assay (ELISA) (R&D Systems, Inc., Minneapolis, MN, USA). Serum leptin levels were measured using an ELISA (R&D Systems, Inc., Minneapolis, MN, USA). Serum VEGF (VEGF) levels were measured using an ELISA (R&D Systems, Inc., Minneapolis, MN, USA).

Twenty-four hour urinary excretion of noradrenalin was also determined at baseline and after 8 weeks, using the chromatographic-colorimetric method.

### Dietary supplement

The subjects received two capsules per day of a dietary supplement or an identical placebo. The product was manufactured by Medestea Research & Production S.p.a., (Torino)—Italy. The composition of the dietary supplement is listed in Table 1. Capsicum extract contains a pungent active ingredient, capsaicin. In this particular dietary supplement, the capsicum extract is micro-encapsulated to improve its tolerability. This innovative method of micro-encapsulation enables the administration of one total dose to be subdivided into hundreds of micro-doses. These micro-doses are spread out evenly over a very extensive area of the intestinal mucosa, thus enabling uniform absorption of the active ingredient, over a pre-established timeframe (6 h). The resulting concentration of the active ingredient in each micro-dose is minimal, thus decreasing the risk of local irritation. Moreover, the tablets of this product are gastro-resistant, in order to avoid any kind of irritation in the oral and gastric areas. Identical capsules for each treatment group were assigned to a subject number according to a coded (AB) block randomization table prepared by an independent statistician. Investigators were blinded to the randomization table, to the code assignments and to the procedure. Compliance to the supplementation regimen was defined as the number of tablets actually taken by each subject, divided by the number of tablets that should have been taken over the course of the study. Adverse events were based on spontaneous reporting by subjects as well as open-ended enquiries by members of the research staff.

**Table 1:** Characteristics of the dietary supplement

Botanical extracts	mg/cpr	mg/die
Active ingredients		
*Camellia sinensis* decaffeinated dried extract, mixed with soya phospholipids	150	300
Microencapsulated oleoresin of *Capsicum annum*	7.5	15
l-Carnitine	150	300
*Fucus vesiculosus* dry extract	56.5	113
*Allium sativum* dried extract	2.5	5
Microencapsulated mint essential oil	2.5	5
*Piper nigrum* dry extract	3	6

### Sample size calculation

The sample size calculation is based on the primary endpoint and on information retrieved from scientific literature [56]. We hypothesized a decrease in free fat mass of 0.5 ± 2.5 kg in the control group and of 2.5 ± 2.5 kg in the experimental group, corresponding to a between-group difference of 0.8 standard deviations (effect size). To show such a difference, with a 2-sided type I error of 5 % and a power of 90 %, 50 patients per group are required. The power would be reduced to 82 % in the presence of a dropout rate of about 20 %. nQuery 4 (Statistical Solutions, Cox, IRL) was used to perform the calculations.

### Statistical analysis

Data were described as mean and standard deviation if continuous, and as counts and percentage if categorical. Pre-post comparisons were carried out within each treatment group using the paired Student *t* test; mean changes over time and 95 % confidence intervals (95 %CI) were calculated. Finally, the changes between the treatment groups were compared using a general linear regression model, adjusting for baseline values. Huber White robust standard errors were calculated. The mean difference between the changes and 95 % CI was recorded. Stata 12 (StataCorp, College Station, TX, USA) was used for these calculations. A 2-sided *p* value < 0.05 was considered to be statistically significant.

## Results

**Fig. 1:**
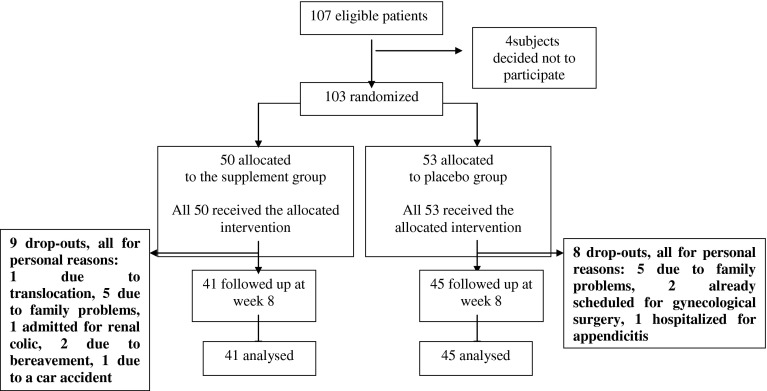
Flow diagram illustrating a clinical trial of a food supplement versus a placebo in the treatment of healthy overweight subjects

One hundred and three overweight subjects were included in the study, out of one hundred and seven eligible participants (Fig. 1). Forty-one patients (26 women; **15** men; aged 43.7 ± 8.5 years with BMI 30.3 ± 3.5 kg/m^2^) were randomized to the supplemented group and 45 (29 women; 16 men; age 40.7 ± 10.2 years and BMI 30.0 ± 2.7 kg/m^2^) to the control group. The baseline characteristics of the supplemented group and the control group are summarized in Table 2, with no relevant differences observed in any of the baseline variables. In addition, there was no significant difference in the baseline characteristics between study completers and drop-outs. The dietary supplement was well tolerated, and there were no reports of any serious adverse events as a result of administration. This finding supports and is consistent with the safety profile that is already widely reported in scientific literature regarding the use of capsaicin and the other plant extracts contained in the formulation [57]. Compliance was completed in both groups and there were no comments from any of the studied patients regarding the content of the supplement he/she was taking, or regarding their perception of having been included in one of the two groups. Changes from baseline were compared between the two treatment arms; these results are summarized in Table 3 (for biochemical and urinary parameters), Table 4 (for metabolic, body composition and QoL parameters) and Table 5 (for anthropometric parameters). A significant difference between treatment groups was observed for the following parameters: insulin levels (*p* = 0.003), insulin resistance (assessed by HOMA (*p* = 0.002) and QUIKI (*p* = 0.007)), leptin/adiponectin ratio (*p* = 0.040), RQ (*p* = 0.008), LDL-cholesterol levels (*p* = 0.031) and urinary norepinephrine levels (*p* < 0.001). No significant differences between the treatment arms were shown for leptin, ghrelin, PCR, REE and adiponectin. Nonetheless, there was a significant decrease in leptin, ghrelin and PCR and an increase in REE (by 120 kcal/die) in the supplemented group but not in the placebo group, whilst adiponectin levels decreased significantly in the placebo group, but not in the supplemented group. Similarly, no significant differences were observed between treatment groups for BMI, fat mass, REE/free fat mass and VEGF. Once again, there was a significant decrease in BMI, fat mass and VEGF and a significant increase in the REE/free fat mass ratio in both groups, with a greater change recorded overall in the supplemented group in comparison to the placebo group. No significant differences were observed neither for any blood chemistry values nor for any of the other anthropometric parameters studied (which all showed a significant decrease from baseline). Finally, no significant differences between the treatment groups were apparent in relation to the sense of satiety, BDI-II and the 2 subscales of the 36-item Short-Form Survey. In the supplemented group, 14 patients (34 %) lost at least 5 % of their body weight, whilst just 10 patients (22 %) from the placebo group fell into this category [Risk difference (IC95 %): 12 % (−7 to 31 %), *p* = 0.24]. Table 6 shows the results of a 3-day weighed-food record of 2 weekdays and 1 weekend day, carried out by all patients from both groups during the first week and the last week of intervention. No significant differences were apparent between the two treatment groups.

**Table 2:** Basal characteristics and biochemical parameters of subjects studied

Variable	Supplemented	Placebo
No. of subjects studied	50	53
No. of females/males	37/13	37/16
No. of drop-outs	9	8
Age (y)	43.7 (±8.5)	40.8 (±10.2)
BMI (kg/m^2^)	30.4 (±3.6)	30.0 (±2.8)
Waist (cm)	98.7 (±11.8)	97.9 (±7.4)
Hips (cm)	109.7 (±8.3)	106.8 (±6.6)
Fat mass (kg)	34.4 (±6.8)	33.3 (±7.3)
Free fat mass (kg)	46.2 (±10.1)	45.5 (±9.7)
Resting energy expenditure (kcal/die)	1483 (±328)	1504 (±295)
Resting energy expenditure/free fat mass	32.5 (±5.7)	35.0 (±1.7)
RQ	0.82 (±0.06)	0.82 (±0.06)
FFAs (mM/L)	0.41 (±0.2)	0.41 (±0.19)
Glycerol (mM/L)	0.15 (±0.05)	0.14 (±0.04)
Adiponectin (ng/mL)	83.63 (±46.26)	83.6 (±53.02)
Leptin (pg/mL)	214.16 (±140.5)	222.68 (±187.5)
Leptin/Adiponectin	3.5 (±3.11)	3.3 (±3.15)
VEGF (pg/mL)	304.75 (±190.42)	332.84 (±228.63)
Ghrelin (pg/mL)	414.58 (±235.25)	395.61 (±275.51)
Urinary norepinephrine (nM/24 h)	40.58 (±24.27)	41.53 (±23.99)

Values are means (±SD) (All randomized patients are included)

**Table 3:** Comparison of biochemical and urinary parameters between the supplemented and placebo groups

Variable Δ	Dietary supplement group mean change (95 % CI)^a^	Placebo group mean change (95 % CI)^a^	Treatment effect mean difference (95 % CI) [adjusted for baseline]	*p* value
Insulin (pmol/L)	1.8 (0.72 to 2.9)	0.11 (−0.88 to 1.1)	17 × 10^−4^ (6 × 10^−4^ to 27 × 10^−4^)	0.003
Homeostatic metabolic assessment index (HOMA)	0.42 (0.14 to 0.71)	0.02 (−0.21 to 0.26)	4 × 10^−4^ (1 × 10^−4^ to 7 × 10^−4^)	0.007
Quantitative Insulin-sensitivity check index (QUICKI)	−0.01 (−0.02 to −0.01)	0.00 (−0.01 to 0.01)	−12 × 10^−6^ (−20 × 10^−6^ to −5 × 10^−6^)	0.002
Leptin/Adiponectin	0.46 (−0.09 to 1.0)	−0.54 (−1.4 to 0.30)	88 × 10^−5^ (3 × 10^−5^ to 173 × 10^−5^)	0.04
LDL-cholesterol (mmol/L)	−4.9 (−10.8 to 0.93)	6.6 (−1.4 to 14.6)	−0.01 (−0.02 to 0.00)	0.013
Urinary norepinephrine (nM/24 h)	−24.6 (−39.6 to −9.6)	5.0 (−3.4 to 13.4)	−0.03 (−0.04 to −0.01)	<0.001
Adiponectin (ng/mL)	4.6 (−1.7 to 10.9)	11.5 (5.8 to 17.2)	−0.01 (−0.01 to 0.00)	0.08
Total cholesterol (mmol/L)	−1.8 (−10.1 to 6.5)	7.2 (−0.58 to 15.1)	−0.01 (−0.02 to 0.00)	0.09
Leptin (pg/mL)	32.6 (9.9 to 55.3)	5.2 (−24.2 to 34.6)	0.03 (0.00 to 0.06)	0.10
Ghrelin (pg/mL)	83.3 (4.8 to 161.8)	0.45 (−85.4 to 86.3)	0.07 (−0.02 to 0.16)	0.14
Apolipoprotein B (g/L)	−8.3 (−16.7 to 0.17)	−1.8 (−8.5 to 4.8)	−0.01 (−0.01 to 0.00)	0.16
FFAs (mM/L)	0.04 (−0.02 to 0.10)	−0.02 (−0.08 to 0.03)	5 × 10^−5^ (−2 × 10^−5^ to 11 × 10^−5^)	0.16
CRP (mg/dL)	0.14 (0.02 to 0.27)	0.08 (−0.04 to 0.19)	5 × 10^−5^ (−3 × 10^−5^ to 13 × 10^−5^)	0.20
Glycaemia (mmol/L)	1.7 (−1.8 to 5.2)	0.42 (−1.7 to 2.5)	1 × 10^−3^ (−2 × 10^−3^ to 4 × 10^−3^)	0.36
Triacylglycerol (mmol/L)	6.8 (−13.5 to 27.1)	−11.6 (−48.2 to 25.0)	0.02 (−0.02 to 0.05)	0.39
HDL-cholesterol (mmol/L)	1.8 (−0.77 to 4.3)	3.0 (0.64 to 5.3)	−1 × 10^−3^ (−4 × 10^−3^ to 2 × 10^−3^)	0.42
Apolipoprotein A1 (g/L)	−1.1 (−11.6 to 9.4)	−3.2 (−13.0 to 6.7)	0.00 (−0.01 to 0.01)	0.42
VEGF (pg/mL)	94.9 (43.3 to 146.5)	86.9 (43.0 to 130.8)	0.02 (−0.03 to 0.07)	0.49
Glycerol (mM/L)	0.00 (−0.02 to 0.01)	−0.01 (−0.02 to 0.01)	−1 × 10^−6^ (−17 × 10^−6^ to 14 × 10^−6^)	0.87
Tot Chol/HDL-Chol	−0.14 (−0.29 to 0.01)	−0.12 (−0.33 to 0.08)	−1 × 10−5 (−23 × 10−5 to 21 × 10−5)	0.90

^a^Within-group changes are calculated as a baseline-final value. So a plus sign (+) corresponds to higher values at baseline (or a decrease during follow-up) and a minus sign (-) to lower values at baseline (therefore indicating an increase during follow-up)

(Variables with statistically significant differences are presented first; the other variables are listed according to the calculated *p* value)

**Table 4:** Comparison of the metabolic, body composition and health-related quality of life parameters studied between the supplemented and placebo groups

Variable Δ	Dietary supplement group mean change (95 % CI)	Placebo group mean change (95 % CI)	Treatment effect mean difference (95 % CI) [adjusted for baseline]	*p* value
RQ	0.04 (−0.01 to 0.09)	−0.04 (−0.09 to 0.01)	8 × 10^−5^ (2 × 10^−5^ to 13 × 10^−5^)	0.008
Resting energy expenditure (kcal)	−120.6 (−220.9 to −20.2)	8.6 (−86.8 to 103.9)	−0.10 (−0.21 to 0.02)	0.10
Fat free mass (kg)	0.36 (−0.03 to 0.76)	−0.13 (−0.66 to 0.40)	4 × 10^−4^ (−1 × 10^−4^ to 10 × 10^−4^)	0.14
Resting energy expenditure/Free fat mass	−3.0 (−5.5 to −0.58)	2.1 (−2.3 to 6.4)	−2 × 10^−3^ (−4 × 10^−3^ to 1 × 10^−3^)	0.22
Fat mass (kg)	2.5 (1.6 to 3.3)	1.9 (1.1 to 2.7)	5 × 10^−4^ (−4 × 10^−4^ to 14 × 10^−4^)	0.24
36-item short-form survey. Mental component subscale (SF-36 MCS)	4.0 (0.68 to 7.4)	1.4 (−2.6 to 5.4)	2 × 10^−3^ (−2 × 10^−3^ to 6 × 10^−3^)	0.24
Beck depression inventory (BDI-II)	3.3 (1.4 to 5.2)	2.3 (0.79 to 3.7)	9 × 10^−4^ (−7 × 10^−4^ to 25 × 10^−4^)	0.26
AUC for satiety VAS	–	–	0.02 (−0.02 to 0.06)	0.36
Android fat (%)	1.8 (1.1 to 2.5)	1.4 (0.59 to 2.2)	4 × 10^−4^ (−5 × 10^−4^ to 13 × 10^−4^)	0.36
36-item short-form survey. Physical component subscale (SF-36 PCS)	5.1 (−0.59 to 10.8)	3.1 (−1.4 to 7.6)	2 × 10^−3^ (−4 × 10^−3^ to 8 × 10^−3^)	0.42
Gynoid fat (%)	1.6 (0.92 to 2.2)	1.4 (0.71 to 2.1)	1 × 10^−4^ (−6 × 10^−4^ to 9 × 10^−4^)	0.70

^a^Within-group changes are calculated as a baseline-final value. Thus, a plus sign (+) corresponds to a higher value at baseline (or a decrease during follow-up) and a minus sign (−) to a lower value at baseline (thus indicating an increase during follow-up)

(Variables with statistically significant differences are presented first; the other variables are listed according to the calculated *p* value)

**Table 5:** Comparison of the anthropometric parameters studied between the supplemented and placebo groups

Variable Δ	Dietary supplement group mean change (95 % CI)^a^	Placebo group mean change (95 % CI)^a^	Treatment effect mean difference (95 % CI) [adjusted for baseline]	*p* value
Weight (kg)	3.1 (2.2 to 4.0)	2.0 (1.1 to 2.8)	9 × 10^−4^ (−1 × 10^−4^ to 20 × 10^−4^)	0.08
BMI (kg/m^2^)	1.1 (0.78 to 1.4)	0.71 (0.39 to 1.0)	33 × 10^−5^ (4 × 10^−5^ to 70 × 10^−5^)	0.08
Waist hip ratio (WHR)	0.00 (0.00 to 0.01)	0.01 (0.00 to 0.03)	−1 × 10^−5^ (−2 × 10^−5^ to 15 × 10^−5^)	0.09
Arm circumference (cm)	0.60 (0.28 to 0.92)	0.59 (0.12 to 1.1)	1 × 10^−4^ (−4 × 10^−4^ to 6 × 10^−4^)	0.65

^a^Within-group changes are calculated as the baseline-final value. Therefore a plus sign (+) corresponds to a higher value at baseline (or a decrease during follow-up) and a minus sign (−) to a lower value at baseline (thus indicating an increase during follow-up)

(Variables with statistically significant differences are presented first; the other variables are listed according to the calculated *p* value)

**Table 6:** A 3-day weighed-food record of 2 weekdays and 1 weekend day, performed during the first week and the final week of intervention

	First week of intervention (dietary supplement)	Final week of intervention (dietary supplement)	First week of intervention (placebo)	Final week of intervention (placebo)
Energy (kJ)	6761 (65.2)	6792 (64.5)	6652 (59.9)	6766 (67.2)
Protein (g); (% energy)	70.8 (4.1); 17.5 (0.9)	70.8 (5.0); 17.5 (1.0)	63.0 (6.3); 15.9 (1.1)	69.3 (5.9); 17.2 (1.0)
Fat (g); (% energy)	48.7 (3.3); 27.2 (2.1)	47.1 (2.9); 26.1 (1.9)	46.9 (4.2); 26.6 (2.0)	52.5 (5.3); 29.2 (2.1)
Saturated fatty acids (g); (% energy)	12.2 (1.5); 6.8 (0.7)	12.8 (2.1); 7.1 (1.0)	12.0 (2.4); 6.8 (0.8)	14.9 (3.0); 8.3 (0.9)
Carbohydrate (g); (%)	238.2 (6.1); 55.3 (1.6)	243.8 (5.7); 56.4 (1.9)	243.8 (7.4); 57.5 (2.0)	231.0 (8.0); 53.6 (2.1)
Complex (g); (%)	152.3 (20.4); 35.3 (3.1)	167.1 (21.8); 38.7 (2.9)	181.7 (22.3); 42.9 (3.0)	165.7 (23.2); 38.5 (4.0)
Simple (g); (%)	85.9 (10.2); 20 (2.5)	76.7 (11.1); 17.7 (2.7)	62.1 (12.5); 14.6 (2.9)	65.2 (9.9); 15.1 (2.4)
Dietary fibre (g)	26.3 (2.9)	26.5 (3.6)	25.8 (4.2)	24.1 (4.3)
Cholesterol (mg)	128.9 (31.7)	178.6 (30.5)	159.2 (32.4)	204.1 (33.0)

Mean (SD); Nutritional evaluation carried out by Carnovale E, Marletta L: “Food composition tables”, Italian National Insitute of Nutrition, Rome 1997 (“Tabelle di composizione degli alimenti”, Istituto Nazionale della Nutrizione, Roma 1997)

## Discussion

In this study, which was carried out on overweight subjects, we observed that the consumption of this combination of bioactive food ingredients was associated with a decrease in insulin resistance and in inflammatory adipokines. In addition, the difference in body composition was greater in the active treatment arm, particularly in relation to the primary endpoint (fat mass), although no statistically significant results were produced. Obesity is now considered to be a condition that facilitates the development of a low-grade inflammatory state characterized by increased plasma levels of pro-inflammatory cytokines and cytokine-like proteins known as adipokines [22], and that one of the consequences of this state of inflammation is the development of insulin resistance [23]. Adipose tissue secretes adipokines, which may be the missing link between insulin resistance and obesity, influencing body weight, glucose and lipid metabolism [58]. Adiponectin has anti-atherogenic, anti-diabetic and anti-inflammatory properties [59]. Increased adiponectin is associated with a lower risk of impaired glucose tolerance and a decrease in the risk of myocardial infarction, and has been proposed as a biomarker of early atherosclerosis [60]. Leptin, a cytokine-like molecule secreted by adipose tissue, regulates adipose mass and body weight by inhibiting food intake and stimulating energy expenditure [61, 62]. Numerous publications suggest leptin as a biomarker for obesity, insulin resistance and Metabolic Syndrome in adults [61, 62]. The leptin-to-adiponectin ratio was recently proposed as a biomarker with the benefits of both indices [63–65]. Although weight and BMI are the best indicators for the patients, a positive outcome must be measured by an improvement in their overall well-being, not just by weight loss alone. An improvement in the patient’s inflammatory state therefore represents a significant positive outcome. The novelty of our study is the fact that overweight subjects taking the dietary supplement obtained a positive metabolic outcome at the end of the study. In fact, when comparing the effect of the dietary supplement with that of the placebo in our study, we observed that consumption of the dietary supplement was associated with a significant decrease in insulin resistance (assessed by HOMA and QUIKI), leptin/adiponectin ratio, RQ and LDL-cholesterol levels. Leptin, ghrelin and CRP significantly decreased in the supplemented group but not in the placebo group, whilst adiponectin levels significantly decreased in the placebo group but not in the supplemented group, although no statistically significant difference between the groups was recorded. This framework constitutes a significant improvement in the metabolic status of the supplemented patients, especially in light of the fact that weight loss does not always lead to an improvement in a patient’s inflammatory state and insulin resistance, as demonstrated by numerous studies [66, 67]. These positive results on metabolic status are probably due to the synergistic action of the bioactive compounds of this dietary supplement, in particular EGCG and capsaicins. Capsaicin, a biologically active compound found in red pepper, has anti-inflammatory activities [68–70] and demonstrates potential benefits for treating obesity and insulin resistance, both in animal models and in clinical studies [71–74]. Our results therefore concur with previous studies in this area [27–29, 32, 72]. Interestingly, the supplemented subjects experienced a significant reduction in RQ. It has also been suggested that the RQ, as a reflection of carbohydrate and fat oxidation, may be a metabolic index which predicts subsequent weight gain [75]. In one study, a higher 24-h RQ (reflecting greater carbohydrate and less fat oxidation) was found to correlate with a greater risk of weight gain, independently of low-energy expenditure [76]. The findings of the Baltimore Longitudinal Study supported this, in that a higher resting RQ correlated with subsequent weight gain, at least amongst lean men [77]. Another reflection of this pattern of energy usage lies in the fact that insulin sensitivity—and, hence, a tendency to greater carbohydrate use—has been reported to produce a greater weight gain in Pima Indians [75] and in individuals who are already obese [78], but not in lean individuals [78]. Another important result of the study is the significant increase in urinary norepinephrine levels, which was observed only in the supplemented group. This urinary parameter was evaluated to produce an indirect assessment of the absorption and effectiveness of the dietary supplement. EGCG can inhibit catechol *O*-methyltransferase, an enzyme involved in the degradation of norepinephrine [79]. Consequently, once released, norepinephrine remains in the synaptic cleft for longer, and provides prolonged stimulation of the adrenergic receptors. Interestingly, a number of compounds extracted from plants—such as capsaicin from pungent spices—can modulate catecholamine release and activity [80].

Finally, another important result of this study was the effect of the dietary supplement on REE; this increased significantly in the supplemented group but not in the placebo group. Studies examining changes in energy requirements following moderate weight loss have shown that active weight loss is associated with a decrease in REE, and that this decrease is disproportionately large in relation to the loss of body tissues [81–83]. Our results show that the dietary supplement, in combination with a low-calorie diet, can counteract this decrease in REE. The results of the study demonstrated that REE increases only in the treated group. This increase in REE is significantly advantageous from a metabolic perspective. Our results concur with the available literature: there is a substantial quantity of data available to substantiate the metabolism-enhancing properties of pepper and green tea [4, 26–30]. As regards appetite, the results of this study confirm that EGCG and capsaicin inhibit appetite effectively [6, 32]. The dietary supplement used in this study is certainly an innovation. We consider that the majority of the results produced by this study can be attributed to the synergistic action of the components. Compared to the results published in previous scientific literature, this supplement is more effective in increasing the basal metabolic rate, improving insulin resistance and inducing favourable changes in plasma inflammatory adipokines.

Our study does have some limitations, the first of which is the relatively short duration of the observation period, although this is similar to the duration of other previous studies carried out on the same substances [6, 8, 27, 37]. A further limitation lies in the fact that we did not test the efficacy of the individual components of the dietary supplement used in the trial, although there is a substantial quantity of scientific literature available relating to the activities of the individual components within this combination of bioactive food ingredients [5–14, 25–38]. Another problem stems from the fact that the personalized weight-loss programme was relatively short, and therefore its efficacy could have overlapped at least partially with the effects of the tested dietary supplement. However, the study had a placebo arm and the placebo subjects also received a personalized weight-loss programme. Changes in biochemical indices and body composition are separate endpoints and were therefore evaluated as such, as projected in the protocol. We might hypothesize that some of the effect seen in one endpoint may be explained by the other. Post-hoc sub-analyses are planned in the future in order to address this, along with other issues.

In conclusion, the results of this study indicate that this combination of bioactive food ingredients could produce significant beneficial actions in the management of overweight-related inflammation, by reducing insulin resistance and inflammatory adipokines.
